# Reviewers The publication of invaluable papers in Cancer Science depends on the prompt, careful review of submitted manuscripts. We would like to thank the following experts for reviewing manuscripts submitted between October 1, 2023 to September 30, 2024

**DOI:** 10.1111/cas.16399

**Published:** 2024-12-02

Reviewer names

Yuichi Abe

Naim Abu‐Freha

Jun Adachi

Shungo Adachi

Ahmad Adawy

Jiyoung Ahn

Kiwamu Akagi

Yu Akagi

Shusuke Akamatsu

Shinya Akatsuka

Yoshiki Akatsuka

Yoshimitu Akiyama

Kazunari Akiyoshi

Kiyohiro Ando

Masashi Ando

Mizuo Ando

Toshinori Ando

Kenjiro Aogi

Hiroyuki Arai

Seiji Arai

Tomio Arai

Yoshiki Arakawa

Hidetaka Arimura

Tomohiro Arita

Ken Asada

Shuhei Asada

Hajime Asahina

Kazuo Asanoma

Kunihiro Asanuma

Tetsuhiko Asao

Yoshinari Asaoka

Eishi Ashihara

William Atiomo

Kotaro Azuma

Eishi Baba

Hideo Baba

Tsukasa Baba

Yoshifumi Baba

Mohamadreza K Bakht

Oluwaseun Adebayo Bamodu

Lyudmila Bel'Skaya

Prateek Bhatia

Sergei Boichuk

Horacio Cabral

Shang Cai

Kaixiang Cao

Carmine Carbone

Joaquim Carreras

Michael Chan

Yongsheng Jason Chan

Jianfeng Chang

Hadrien Charvat

Liang Chen

Yang Chen

Zhiyu Chen

Pu Cheng

Ying‐Cheng Chiang

Hideki Chiba

Natsuko Chiba

Shigeru Chiba

Kazuaki Chikamatsu

Tatsuyuki Chiyoda

Weimin Ci

Samuel Cohen

Laura Cortesi

Karen Crasta

Haruko Daga

Takiko Daikoku

Shingo Dan

Santanu Dasgupta

Thomas Daubon

Ayako Demachi‐Okamura

Yosuke Demizu

Jie Dong

Dan Duda

Hidetoshi Eguchi

Takanori Eguchi

Shogo Ehata

Elamin H Elbasha

Yutaka Endo

Daisuke Ennishi

Hideki Enokida

N. E. Ezinne

Dong Fang

Weijia Fang

Fan Feng

Hirofumi Fujii

Satoshi Fujii

Shin‐Ichiro Fujii

Atsushi Fujimura

Takao Fujisawa

Mitsuhiro Fujishiro

Teruaki Fujishita

Kazutoshi Fujita

Hiroshi Fujiwara

Naoto Fujiwara

Yukio Fujiwara

Tomoya Fukawa

Akihisa Fukuda

Shinji Fukuda

Shigetomo Fukuhara

Keisuke Fukui

Hidenori Fukuoka

Noriyoshi Fukushima

Itoh Fumiko

Ryo Funayama

Tatsuhiko Furukawa

Tatsuhiko Furukawa

Toru Furukawa

Yusuke Furukawa

Mikio Furuse

Mitsuru Futakuchi

Ping Gao

Yuzhen Gao

Min Gi

Hidemasa Goto

Osamu Gotoh

Adam L. Green

Hiroshi Haeno

Shin Hamada

Tsuyoshi Hamada

Yoichiro Hamamoto

Junzo Hamanishi

Yasushi Hamaya

Ichiro Hanamura

Megumi Hara

Tomoaki Hara

Hiroshi Harada

Kenji Harada

Naoaki Harada

Yoichiro Harada

Koji Haratani

Koichiro Haruki

Ari Hashimoto

Naoya Hashimoto

Shinichi Hashimoto

Tadayoshi Hashimoto

Nobuhiro Hata

Ryusuke Hatae

Shigetsugu Hatakeyama

Etsuro Hatano

Naoko Hattori

Fumihiko Hayakawa

Yoku Hayakawa

Hidetoshi Hayashi

Hideyuki Hayashi

Yoshihiro Hayashi

Masaharu Hazawa

Yalda Hekmatshoar

Tokimasa Hida

Fumi Higuchi

Shinjiro Hino

Kunihiko Hinohara

Takao Hinoi

Hideyo Hirai

Tomoko Hirano

Nobuyoshi Hiraoka

Akira Hirasawa

Eishu Hirata

Hidenari Hirata

Hiroshi Hirata

Kenji Hirata

Kenro Hirata

Makoto Hirata

Yoshihiko Hirohashi

Shuichi Hironaka

Kenzo Hiroshima

Yuichi Hisamatsu

Shigeo Hisamori

Asahi Hishida

Takaki Hiwasa

Eiso Hiyama

Shohei Honda

Junbo Hong

Tsunaki Hongu

Naoko Honma

Yoshitaka Honma

Megumi Hori

Kuniko Horie

Masafumi Horie

Keisuke Horiuchi

Naoki Hosen

Daisuke Hoshino

Naoko Hosono

Satoyo Hosono

Yasuyuki Hosono

Noriko Hosoya

Katsuyuki Hotta

Qingjiang Hu

Guoquan Huang

Haojie Huang

Lizhen Huang

Zhenyu Huo

Ichinosuke Hyodo

Daisuke Ichikawa

Tomonaga Ichikawa

Masashi Idogawa

Hiroshi Igaki

Yoshito Ihara

Yuichi Iida

Hisashi Iizasa

Hideaki Ijichi

Kazuhiro Ikeda

Satoshi Ikeda

Shinya Ikematsu

Yoichi Imai

Ryu Imamura

Toshihiko Imamura

Yorihisa Imanishi

Yoshimi Imawari

Fuyuki Inagaki

Daichi Inoue

Masahiro Inoue

Masayoshi Inoue

Takahiro Inoue

Yasumichi Inoue

Takashi Inozume

Taiki Isei

Seiichiro Ishihara

Akira Ishikawa

Eiichi Ishikawa

Nobuhisa Ishikawa

Shigeo Ishikawa

Yuichi Ishikawa

Takatsugu Ishimoto

Yosuke Ishitsuka

Kota Itahashi

Hiroaki Itamochi

Hidemi Ito

Ken‐Ichi Ito

Kosei Ito

Takahiro Ito

Yuri Ito

Shinji Itoh

Junji Itou

Kazuya Iwabuchi

Yasuo Iwadate

Masao Iwagami

Yoshifumi Iwagami

Moriya Iwaizumi

Atsushi Iwama

Eiji Iwama

Takeshi Iwasaki

Shintaro Iwata

Yasumasa Iwatani

Takeshi Iwaya

Naoki Izawa

Koji Izutsu

Baoan Ji

Xingming Jiang

Ze Jin

Natini Jinawath

Yoshikazu Johmura

Hemant Joshi

Tetsuya Kadonosono

Hiroshi Kagamu

Hidenori Kage

Yuki Kagoya

Taisuke Kajino

Hiroaki Kajiyama

Anna Kakehashi

Nobuyuki Kakiuchi

Yoichiro Kamatani

Takashi Kamei

Walied Kamel

Yasuhiko Kamikubo

Masashi Kanai

Yoshikatsu Kanai

Takayuki Kanaseki

Hiroaki Kanda

Shuzo Kaneko

Yonehiro Kanemura

Takahiro Karasaki

Yoshinobu Kariya

Kennosuke Karube

Kazuo Kasahara

Hiroaki Kasashima

Akiyoshi Kasuga

Hiroaki Kataoka

Kazuhiro Katayama

Koji Kato

Minoru Kato

Renpei Kato

Taigo Kato

Takuma Kato

Yukinari Kato

Hiroto Katoh

Keisuke Katsushima

Kimihito Kawabata

Manabu Kawada

Kosuke Kawaguchi

Yo Kawaguchi

Kohichi Kawahara

Masataka Kawai

Taketo Kawai

Akinori Kawakami

Hisato Kawakami

Daisuke Kawakita

Tomokazu Kawaoka

Yoshihiro Kawasaki

Atsunari Kawashima

Daisuke Kawauchi

Akihito Kawazoe

Masahito Kawazu

Tanaka Kazuhiro

Hiroyasu Kidoya

Eiji Kikuchi

Shojiro Kikuchi

Toyone Kikumori

Junichi Kikuta

Takahiro Kimura

Ichiro Kinoshita

Manabu Kinoshita

Connor J Kinslow

Hiroshi Kitajima

Shunsuke Kitajima

Kazuhisa Kitami

Hidemitsu Kitamura

Hiroshi Kitamura

Yotaro Kitano

Hiroki Kiyokawa

Nobutaka Kiyokawa

Tamotsu Kiyoshima

Kazuma Kiyotani

Zdeněk Kleibl

Eiji Kobayashi

Hiroshi Kobayashi

Hiroya Kobayashi

Hisataka Kobayashi

Kazuma Kobayashi

Satoshi Kobayashi

Shogo Kobayashi

Takahiko Kobayashi

Takashi Kobayashi

Yoshihisa Kobayashi

Yukari Kobayashi

Vitaly Kochin

Takahiro Kodama

Satoshi Kofuji

Hironori Koga

Tomoyuki Koga

Yoshikatsu Koga

Takahiro Kogawa

Yasunori Kogure

Yasuhiro Koh

Kenichi Kohashi

Susumu Kohno

Shinji Kohsaka

Shiro Koizume

Motohiro Kojima

Takahiro Kojima

Yasuhiro Kojima

Yasushi Kojima

Yu‐Ichiro Koma

Masaaki Komatsu

Yoshito Komatsu

Daisuke Komura

Kazumasa Komura

Shunsuke Kon

Jumpei Kondo

Takeshi Kondo

Hiroshi Kondoh

Akimitsu Konishi

Masamitsu Konno

Chihaya Koriyama

Naohiko Koshikawa

Shinji Kosugi

Fujimoto Kosuke

Ai Kotani

Daisuke Kotani

Nobuji Kouno

Taku Kouro

Keita Kouzu

Shohei Koyama

Takahiro Koyanagi

Yuriko Koyanagi

Terufumi Kubo

Kazunori Kubota

Shimpei Kubota

Aya Kuchiba

Yotaro Kudo

Nilakshi Kulathunga

Shogo Kumagai

Ranjith Kumavath

Kei Kunimasa

Morito Kurata

Ryota Kurimoto

Hiroshi Kurita

Sei Kuriyama

Junya Kuroda

Koji Kurose

Kazuhiko Kurozumi

Yoshihiro Kushihara

Shigeru Kusumoto

Takeshi Kuwata

Satoru Kyo

Zhicheng Lai

Tobias Langenberg

Ming‐Shyue Lee

Jinluan Li

Lianbo Li

Yan‐Ruide Li

Yu Li

Zhi Li

Zhuan Li

Xinhua Liang

Susan W. Liebman

Ming‐Fong Lin

Shaoqiang Lin

Haiou Liu

Shirong Liu

Xiaofeng Liu.

Aaron C. Logan

Peng Luo

Fuquan Ma

Hidenori Machino

Mitsuhiro Machitani

Shin Maeda

Takahiro Maeda

Tomohiko Maehama

Ryoichi Maenosono

Nako Maishi

Teruhiko Makino

Hideki Makinoshima

Hideki Makishima

Shinichi Makita

Yoshiaki Maru

Dai Maruyama

Reo Maruyama

Tetsuo Mashima

Mari Masuda

Muneyuki Masuda

Takaaki Masuda

Yohei Masugi

Kenta Masui

Toshiiki Masuishi

Chikahide Masutani

Mitsuko Masutani

Daisuke Matsubara

Satoru Matsuda

Tomohiro Matsuda

Kosei Matsue

Nobuhisa Matsuhashi

Akihiko Matsumine

Akinobu Matsumoto

Hiromi Matsumoto

Kazuhiro Matsumoto

Koji Matsumoto

Shingo Matsumoto

Shinji Matsumoto

Tomonori Matsumoto

Yoshihiro Matsumoto

Yoshihisa Matsumoto

Noriomi Matsumura

Takashi Matsunaga

Keisuke Matsusaka

Juntaro Matsuzaki

Shinji Mii

Hirokazu Miki

Kosuke Mima

Daisuke Minami

Yosuke Minami

Yoji Minamishima

Toshinari Minamoto

Lisa Mirabello

Sheefa Mirza

Yuichi Mitobe

Makoto Mitsunaga

Shuichi Mitsunaga

Masahiko Miura

Etsuko Miyagi

Yohei Miyagi

Kosuke Miyai

Makito Miyake

Mototaka Miyake

Shin‐Ichi Miyatake

Eisaku Miyauchi

Masashi Miyauchi

Makoto Miyazaki

Hiroaki Miyoshi

Yasuo Miyoshi

Atsushi Mizokami

Yusuke Mizukami

Kazunori Mizuno

Ryuichi Mizuno

Yasuyuki Mizutani

Mehdi Mohammadi

Yukihide Momozawa

Seiichi Mori

Kohji Moriishi

Masahiro Morise

Ken Morita

Toshiro Moroishi

Shinichiro Motohashi

Noriko Motoi

Ping Mu

Ken‐Ichi Mukaisho

Toru Mukohara

Wataru Munakata

Rafaela Muniz De Queiroz

Jordi Muntane

Junko Murai

Kazuhiro Murakami

Kosuke Murakami

Ryusuke Murakami

Yoshiki Murakumo

Ayako Muraoka

Daisuke Muraoka

Kei Muro

Manabu Muto

Michihiro Mutoh

Genta Nagae

Masayuki Nagahashi

Hiroaki Nagano

Osamu Nagano

Yoshihiro Nagao

Koji Nagaoka

Kazunori Nagasaka

Misako Nagasaka

Hiroaki Nagashima

Kengo Nagashima

Yasunobu Nagata

Satoshi Nagayama

Taku Naiki

Aya Naiki‐Ito

Hisamichi Naito

Yuji Naito

Yutaka Naito

Tasuku Nakabori

Ichiro Nakachi

Hayato Nakagawa

Masahiro Nakagawa

Masao Nakagawa

Yousuke Nakai

Yu‐Ichiro Nakajima

Harumi Nakamura

Masao Nakamura

Motoki Nakamura

Ritsuko Nakamura

Takafumi Nakamura

Toru Nakamura

Yoshiaki Nakamura

Eiji Nakano

Hiroyasu Nakano

Takashi Nakaoku

Takuma Nakatsuka

Izuma Nakayama

Joji Nakayama

Jun Nakayama

Jun Nakayama

Mizuho Nakayama

Tomio Nakayama

Yozo Nakazawa

Shinichi Namba

Takeshi Namekawa

Takeshi Namiki

Yasuhito Nannya

Shintaro Narita

Manabu Natsumeda

Atsushi Niida

Keisuke Nimura

Kiichiro Ninomiya

Jun Nishida

Yuichiro Nishida

Makiya Nishikawa

Yuji Nishikawa

Momoko Nishikori

Tatsunori Nishimura

Hiroshi Nishina

Takashi Nishina

Hitomi Nishinakamura

Yoshikazu Nishino

Mari Nishio

Yasuhiko Nishioka

Michiru Nishita

Hideki Nishitoh

Shin Nishiumi

Naoyuki Nishiya

Atsuya Nishiyama

Hironari Nishizawa

Satoshi Nishizuka

Takehiro Noda

Naoyuki Nogami

Satoshi Nojima

Masashi Nomura

Sachiyo Nomura

Kisato Nosaka

Wataru Obara

Yuki Obata

Daisuke Obinata

Mieko Ochi

Toshiki Ochi

Sadahisa Ogasawara

Dai Ogata

Atsushi Ogawa

Mikako Ogawa

Shuji Ogino

Takashi Ohama

Toshiaki Ohara

Akihiro Ohashi

Kadoaki Ohashi

Hiroto Ohguchi

Tomokazu Ohishi

Fumiharu Ohka

Takayuki Ohkuri

Shinobu Ohnuma

Nobumichi Ohoka

Kenji Ohshima

Takahito Ohshiro

Takao Ohtsuka

Sumio Ohtsuki

Takahiro Oike

Yusuke Oji

Hidenori Ojima

Koji Okabayashi

Ryuhei Okada

Naohiro Okano

Susumu Okano

Yasumasa Okazaki

Eiji Oki

Yukari Okita

Akihiko Okuda

Yoshinaga Okugawa

Yusuke Okuma

Kazuhiro Okumura

Shintaro Okumura

Tracy O'Mara

Yasushi Onishi

Hiroaki Ono

Takashi Onoe

Masahiro Onozawa

Mamiko Onuki

Kunishige Onuma

Akira Orimo

Mitsuhiko Osaki

Takahiro Osawa

Tsuyoshi Osawa

Hiroko Oshima

Hiroki Osumi

Ryohei Otani

Kota Ouchi

Yuko Oya

Shuji Ozaki

Toshinori Ozaki

Yukinori Ozaki

Isao Oze

Maierdan Palihati

Cheng Pan

Jae‐Hyun Park

Mi Kyung Park

Samuel Pena‐Llopis

Jesus Perez‐Losada

Krzysztof Piersiala

Xiaowei Qi

Xian‐Yang Qin

Marika Quadri

Erna Raja

Vivek M Rangnekar

Karthikeyan Ramalingam

Susumu Rokudai

Mirian Romitti

Arya Roy

Ryota Sada

Ken Saijo

Ayumi Saito

Motonobu Saito

Takuro Saito

Toshiyuki Saito

Yasuhiro Saito

Yasuyuki Saito

Yuki Saito

Masao Saitoh

Masakiyo Sakaguchi

Yusuke Sakaguchi

Kazuko Sakai

Yoshio Sakai

Jun Sakakibara‐Konishi

Naoya Sakamoto

Shinichi Sakamoto

Takeharu Sakamoto

Tomohiro Sakamoto

Tomohisa Sakaue

Yukimi Sakoda

Yuji Sakuma

Hideyuki Sakurai

Abdu Salam

Masashi Sanada

Takaomi Sanda

Elumalai Sanniyasi

So‐Ichiro Sasaki

Takaaki Sasaki

Yasushi Sasaki

Megumi Sasatani

Akira Sato

Hiroki Sato

Kuniaki Sato

Mitsuo Sato

Shinya Sato

Tatsuhiro Sato

Yasuyoshi Sato

Yukiko Sato

Yusuke Sato

Yatushi Satoh

Takeshi Sawada

Masahiro Seike

Masau Sekiguchi

Masayuki Sekine

Shiqian Shen

Akiko Shibata

Atsushi Shibata

Norihito Shibata

Tomoyuki Shibata

Yuji Shibata

Hirohiko Shibayama

Makoto Shibutani

Tadahiko Shien

Kiyoto Shiga

Kazuyuki Shimada

Shu Shimada

Tetsuro Shimakami

Teppei Shimamura

Taichi Shimazu

Yutaka Shimazu

Hiroki Shimizu

Kazuya Shimoda

Shinji Shimoda

Tatsunori Shimoi

Akihiko Shimomura

Yuji Shimura

Kato Shinichiro

Seiichi Shinji

Keiko Shinjo

Miki Shinohara

Yasushi Shintani

Daisuke Shiokawa

Takayuki Shiomi

Masaki Shiota

Ken Shirabe

Kouya Shiraishi

Takehito Shukuya

Fusheng Si

Jerson L. Silva

Siro Simizu

Makoto Sohda

Eric Solary

Kenbun Sone

Masahiro Sonoshita

Lena Specht

Le Su

Kenichi Suda

Yusuke Suenaga

Kokichi Sugano

Narushi Sugii

Keishi Sugimachi

Mikio Sugimoto

Daisuke Sugiyama

Eri Sugiyama

Hiromi Sugiyama

Kentaro Suina

Hidetoshi Sumimoto

Dan Sun

Yuxiang Sun

Shigeaki Sunada

Yu Sunakawa

Yoshiaki Sunami

Takeshi Susukida

Ayako Suzuki

Hidefumi Suzuki

Hiromichi Suzuki

Hiroshi I Suzuki

Hiroyuki Suzuki

Kei Suzuki

Miho Suzuki

Norio Suzuki

Rei Suzuki

Takafumi Suzuki

Takeshi Suzuki

Toshihiro Suzuki

Sho Tabata

Takahiro Tabuchi

Masatoshi Tagawa

Ayumi Taguchi

Ayumu Taguchi

Kenichi Taguchi

Ken Tajima

Kohichi Takada

Mamoru Takada

Satoshi Takagi

Takayuki Takahama

Akiko Takahashi

Hayato Takahashi

Hirotaka Takahashi

Ken Takahashi

Masanobu Takahashi

Naoto Takahashi

Nobuaki Takahashi

Ryou‐U Takahashi

Satoshi Takahashi

Takuto Takahashi

Tsuyoshi Takahashi

Atsushi Takahashi‐Kanemitsu

Manabu Takamatsu

Hirokazu Takami

Ken Takasawa

Shinji Takasu

Kenichi Takayama

Haruna Takeda

Kazuyoshi Takeda

Masayuki Takeda

Norihiko Takeda

Hiroyuki Takei

Toshihiko Takeiwa

Masataka Takenaka

Hideyuki Takeshima

Hiroya Takeuchi

Shinji Takeuchi

Yasuto Takeuchi

Toshiro Takezaki

Tetsuro Taki

Hidehiko Takigawa

Nagio Takigawa

Shuji Takiguchi

Keiyo Takubo

Hideto Tamura

Ryo Tamura

Takaaki Tamura

Hiroki Tanabe

Masahiko Tanabe

Atsushi Tanaka

Hajime Tanaka

Ichidai Tanaka

Kentaro Tanaka

Kousuke Tanaka

Minoru Tanaka

Miwa Tanaka

Nobuyuki Tanaka

Noritaka Tanaka

Shota Tanaka

Yosuke Tanaka

Hailin Tang

Masaji Tani

Tomohiko Taniai

Hiroya Taniguchi

Kohei Taniguchi

Koji Taniguchi

Toshiyasu Taniguchi

Chizu Tanikawa

Akihide Tanimoto

Keiji Tanimoto

Yukari Taniyama

Junko Tanizaki

Kayoko Tao

Etsu Tashiro

Hironori Tashiro

Satoshi Tashiro

Keisuke Tateishi

Kensuke Tateishi

Hiro Tatetsu

Daisuke Tatsuda

Yasutoshi Tatsumi

Kenji Tatsuno

Hend M. El Tayebi

Naoki Terada

Seitaro Terakura

Koji Teramoto

Takeshi Terashima

Naoki Teraya

Yasuhito Terui

Khin Khin Tha

Martin K. Thomsen

Hua Tian

Zhongxian Tian

Yuji Toiyama

Yoshihisa Tokumaru

Fuminori Tokunaga

Shuta Tomida

Yoshito Tomimaru

Arata Tomiyama

K. Tomizawa

Takashi Toya

Masashi Toyoda

Markus Tschurtschenthaler

Shoma Tsubota

Benio Tsuchiya

Naoto Tsuchiya

Takemasa Tsuji

Tomohide Tsukahara

Hirotake Tsukamoto

Tetsuya Tsukamoto

Tadasuke Tsukiyama

Yoshinori Tsukumo

Ryouichi Tsunedomi

Takaaki Tsunematsu

Toyonori Tsuzuki

Yusuf Turkoz

Shinya Uchino

Hibiki Udagawa

Keiko Udaka

Yutaka Ueda

Satoshi Ueha

Yuji Uehara

Hiroji Uemura

Motohide Uemura

Sayaka Ueno

Takayuki Ueno

Hiroyuki Uetake

Takashi Umehara

Masanari Umemura

Shigeki Umemura

Fumihiko Urabe

Hiroshi Ureshino

Hirokazu Usui

Yoshiaki Usui

Kensuke Usuki

Mai Utada

Caterina Vaghi

Marco De Velasco

R Verma

Alessandro De Vita

Keiko Wada

Satoshi Wada

Yuichi Wakabayashi

Jianliu Wang

Kai Wang

Qun Wang

Wei Wang

Yuexiang Wang

Keisuke Watanabe

Reiko Watanabe

Satoshi Watanabe

Takashi Watanabe

Yukihide Watanabe

Fei Wu

Peng Wu

Xuan Wu

Yifei Xing

Yang Xu

Tomonori Yaguchi

Keiichi Yamada

Tadaaki Yamada

Takeshi Yamada

Wataru Yamagami

Makoto Yamagishi

Hiroki Yamaguchi

Ken Yamaguchi

Kiyoshi Yamaguchi

Motoko Yamaguchi

Taiki Yamaji

Hideki Yamamoto

Keisuke Yamamoto

Keita Yamamoto

Mizuki Yamamoto

Seiji Yamamoto

Shun Yamamoto

Yuki Yamamoto

Yusuke Yamamoto

Masakatsu Yamashita

Satoshi Yamashita

Taro Yamashita

Tatsuya Yamashita

Takahiro Yamauchi

Manabu Yamazaki

Masaya Yamazaki

Jinsong Yan

Soich Yanamoto

Hiromu Yano

Jun Yasuda

Takahiko Yasuda

Koya Yasukawa

Jun‐Ichirou Yasunaga

Lin Ye

Ji‐Ye Yin

Jianming Ying

Jieer Ying

Kiyotaka Yoh

Akira Yokoi

Sana Yokoi

Hajime Yokota

Kimio Yonesaka

Hiroshi Yoshida

Kenichi Yoshida

Kosuke Yoshida

Noriaki Yoshida

Tatsuya Yoshida

Yoshio Yoshida

Kosuke Yoshihara

Tetsuro Yoshimaru

Yasuhiro Yoshimatsu

Akihide Yoshimi

Katsuhiro Yoshimura

Kiyoshi Yoshimura

Michio Yoshimura

Kiyoshi Yoshino

Kenichi Yoshioka

Yusuke Yoshioka

Hideyuki Yoshitomi

Mitsushiro Yuasa

Kosuke Yusa

Natalia Zeber‐Lubecka

Robert Zeiser

Sadamoto Zenda

Yoshitaka Zenke

Cheng Zhan

Baihao Zhang

Chong‐Jing Zhang

Dingxiao Zhang

Yong Zhang

Lian‐Mei Zhao

Xianda Zhao

Shaoquan Zheng

Jingying Zhou

Guiquan Zhu

Minghua Zhu

Yuyan Zhu

Zhenjian Zhuo

